# Self-Knowledge and Risk in Stratified Medicine

**DOI:** 10.1080/20502877.2017.1314889

**Published:** 2017-05-18

**Authors:** Joshua Hordern

**Affiliations:** ^a^ Faculty of Theology and Religion, Harris Manchester College, Oxford Healthcare Values Partnership, University of Oxford, Oxford, UK

**Keywords:** self-knowledge, risk communication, values, behaviour change, compassion, stratified medicine

## Abstract

This article considers why and how self-knowledge is important to communication about risk and behaviour change by arguing for four claims. First, it is doubtful that genetic knowledge should properly be called ‘self-knowledge’ when its ordinary effects on self-motivation and behaviour change seem so slight. Second, temptations towards a reductionist, fatalist, construal of persons’ futures through a ‘molecular optic’ should be resisted. Third, any plausible effort to change people's behaviour must engage with cultural self-knowledge, values and beliefs, catalysed by the communication of genetic risk. For example, while a Judaeo-Christian notion of self-knowledge is distinctively theological, people's self-knowledge is plural in its insight and sources. Fourth, self-knowledge is found in compassionate, if tense, communion which yields freedom from determinism even amidst suffering. Stratified medicine thus offers a newly precise kind of humanising health care through societal solidarity with the riskiest. However, stratification may also mean that molecularly unstratified, ‘B’ patients’ experience involves accentuated suffering and disappointment, a concern requiring further research.

This paper considers why and how self-knowledge is important to the communication of risk. In an illuminating lecture at the launch of the University of Oxford's Centre for Personalised Medicine in November 2013, Prof Peter Donnelly jokingly remarked that the declining cost of DNA sequencing had made it possible to ‘Know thyself… cheaply’. He attributed the adage ‘Know thyself’ to the bible, suggesting that the biblical authors did not anticipate this use of their words (Donnelly 2013).

This of course would be true if the saying was biblical. But it is not. Self-knowledge is a major theme of the Hebrew and Christian Scriptures and is developed in ways which distinctively shape human attitudes to God, the future, suffering and bodily experience. But the saying is Ancient Greek in its sources, most famously inscribed in the Temple of Apollo at Delphi. It has inspired much reflection, including an emphasis on sober humility: that one ought not to boast that one is or knows more than one in fact is or does in fact know, attitudes which science at its best embodies but which hype can distort.

Humility in self-knowledge is also a theme of Hebrew Scripture, associated with loving fellowship with God.

He has told you, O mortal, what is good;

and what does the Lord require of you

but to do justice, and to love kindness,

and to walk humbly with your God? *(Micah 6:8)*

Psalm 139 gives more insight into the source of such self-knowledge:

**13** For you created my inmost being;

you knit me together in my mother's womb.

**14** I praise you because I am fearfully and wonderfully made;

your works are wonderful,

I know that full well.

**15** My frame was not hidden from you

when I was made in the secret place,

when I was woven together in the depths of the earth.

**16** Your eyes saw my unformed body;

all the days ordained for me were written in your book

before one of them came to be.

**17** How precious to me are your thoughts, God!

How vast is the sum of them! (*Ps 139:13–17)*

For the Psalmist, self-knowledge is distinctively theological: it depends on being known *by* God: coming into being *through* God; pre-natal communion *with* God; the sustaining providence *of* God; and trusting for the future ordained *in* God, that is, in his ‘book’, in which the Psalmist's days are written. The Psalmist does not know himself except through God's knowledge in these domains.

But people's self-knowledge is diverse in at least two ways. On the one hand, the Psalmist varies in the quality of his self-knowledge. He goes on in Psalm 139:

**23** Search me, God, and know my heart;

test me and know my anxious thoughts.

**24** See if there is any offensive way in me,

and lead me in the way everlasting.

He knows that he is anxious and he requires God to find out if there are ‘offensive’ ways within him. Whatever one makes of this mode of interrelation between God and humanity, the Psalmist's concerns bespeak a confusion of thoughts and lack of clear insight in self-knowledge.

On the other hand, in a plural society, even if a general claim about confusion and lack of insight is minimally correct, many do not and will not know themselves as the Psalmist does, as ‘fearfully and wonderfully made’ by a God, or at least not the God of the Psalms. And that diversity presses on an important issue: how *varieties* of self-knowledge relate to perception of risk and behaviour change — how different forms of self-knowledge shape people's perception of probabilities regarding their future well-being and how they then respond.

The UK government's Chief Scientific Advisor's 2014 Annual Report echoed this concern by examining the interplay of innovation, risk and self-understanding. It found that improved communication about risk between specialists and the public could be partially achieved through engagement with the values, beliefs and cultural commitments of the public — or rather the many publics which make up plural, democratic societies (UK Government Office for Science 2014). Such a claim makes interdisciplinary approaches to risk a vital focus for research and policy. Theology, religion and philosophy, alongside social sciences, are well-placed to help with this endeavour. Four remarks on self-knowledge help to spell out what is important here.

## Self-knowledge, motivation and action

First, self-knowledge concerns the interrelation of motivation and action. In March 2016, the BMJ featured a systematic review and meta-analysis of studies examining behaviour change (diet, screening, exercise, etc.) following the communication of DNA-based risk estimates. The conditions covered were diabetes, heart disease, cancers of various sorts, Alzheimer's and obesity.

It found the following:

Expectations that communicating DNA based risk estimates changes behaviour is [sic] not supported by existing evidence. [The] results do not support use of genetic testing or the search for risk-conferring gene variants for common complex diseases on the basis that they motivate risk-reducing behaviour. (Hollands *et al.* 2016, p. 1)

The paper suggests that communication of DNA-based risk estimates makes no major difference to patients’ behaviour; but nor does such communication paralyse patients with a sense of genetic fatalism about the future. Instead, it makes only a very slight difference to behaviour if any at all. The research has some acknowledged weaknesses but broadly reaffirms what other studies have found. These findings raise deeper questions: should genetic knowledge properly be called ‘self-knowledge’ when its ordinary effects on self-motivation are so slight? The salutation of the direct to consumer DNA service 23andme — ‘Welcome to *you*’ (emphasis added) — seems even less plausible in this light. And even if self-knowledge is partially genetic, how much does it have to do with motivation and behaviour as compared to other forms of self-knowledge? How is a person's self-knowledge formed by how I see my identity in relation to the future, what I value and believe, what I ought to do, with whom and why? Answering these questions seems central to improving risk communication and behaviour change in personalised genomic medicine.

## Self-knowledge and the future

Therefore, second, the connection between self-knowledge and the future requires examination. Physician Peter Heusser comments that an ‘almost exclusively molecular and biological concept of “personalisation” leads to a strong connotation — if not to a *de facto* identification — of “person” with the molecular set-up of an individual's physical body. This is a pitfall which ought to be avoided’ (2015, p. 77). Similarly, social scientists Novas and Rose talk disapprovingly of ‘the rewriting of personhood at a genetic level and its visualisation through a “molecular optic”’ (2000, p. 485).

What is the pitfall? It concerns self-knowledge; and it has a double depth: first, an identification of the person through a ‘molecular optic’ reductively obscures or distracts attention from environmental and psycho-social dimensions of persons’ healthcare; but second, this reductionism can slip down into a fatalistic paralysis because of a deterministic interpretation of the genome: perhaps utilising a blueprint or programme metaphor of causation; rather than a systems approach which considers all the influences — physiological and patho-physiological — at a whole organism level; let alone the environmental and psycho-social factors which shape a person (Rehmann-Sutter 2010).

But how big a threat is a reductionist and fatalist sense of self-knowledge? Well perhaps not very great for clinicians and scientists. It seems plausible that stratification of individuals into sub-groups according to genetic characteristics and risks would work *against* the reduction of any particular individual to those characteristics and risks. For genomics-based ‘personalised medicine’ is self-consciously not *typically* about treating persons in their *individuality* but rather as members of sub-groups stratified according to a very narrow specification.

Moreover, clinicians and scientists know about environmental and psycho-social dimensions of health. Note Mathias Wirth's warning against being ‘unjust to physicians’ in a rush to ‘blame a molecular-oriented medicine’ for a reductive form of personalisation (Wirth 2015, p. 65). An accusation of reductionism creates a helpful straw man for medical ethicists seeking a target for criticism. But such a representation seems likely to be unjust to physicians and scientists who may be rather conscious of the difference between individuals and groups precisely because risk has to do with populations rather than with individuals per se.

So these factors should in principle save physicians and others from tumbling into the reductionist-fatalist pitfall. But there is always temptation: the tendency towards certain kinds of knowledge becoming unduly dominant over time, and for knowing and willing to become unhinged. An interview study of German physicians in stratified medicine cites the worry that clinicians will become ‘administrators of markers. That the art of healing and — What is a human? What is the end of life? How to deal with the illness? — that this gets into the background.’ It is suggested that ‘due to the increased differentiation of expertise, it is likely that a holistic physician–patient relationship will be constantly eroded’ and that ‘treatment could be constantly generalised due to increasing standardisation and usage of molecular biomarkers, with the result that the focus of the treatment lies on the specific parameter of the illness and body functions instead of on the patient’ (Wäscher *et al.* 2015, pp. 155, 156).

If this is the case with physicians, how much more of a temptation would it be for employees of pharmaceutical, data-trading and insurance companies, distanced from the disciplining experience of clinical encounter to which Tim Maughan refers in the opening paper of this journal issue (Maughan, this volume).

What about patients? Fatalism about the future was not suggested by the BMJ study. Patients do not identify themselves with genetic predispositions or at least not sufficiently to change their behaviour. Nonetheless, it may be that a reductionist, fatalist view of genetics remains highly influential.

## Self-knowledge, risk and values

This leads to a third issue: how self-knowledge concerns risk and values. The presentation of risk in terms of statistics may shape people's self-knowledge, perhaps profoundly. Communicating risk in terms of the quantification of probabilities requires skill. The very extensive literature on this topic cannot be examined in detail here. One seemingly consensus observation arising from it is that visual representations/pictographs/factboxes rather than or alongside bare numbers are likely to be more effective in enabling better understanding even if not behaviour change; similarly, absolute risk rather than relative risk is easier to grasp. A robust interchange about the use of relative and absolute risks with respect to prostate cancer in the BMJ shows how politically charged this matter is, with accusations of charities having a special interest in promoting universal screening even if there is strong evidence to suggest screening does more harm than good (Gigerenzer 2016).

But language and metaphor are also important. Consider the difference between ‘programme’ and ‘system’ mentioned earlier. In the context of predicting cancer risk, for example, ‘programme’ may suggest the idea that ‘[I have] a genetic fault’; but ‘system’ may suggest ‘an elevated likelihood that the body under certain circumstances can get cancer’ (Rehmann-Sutter 2010, p. 25). These two may shape self-knowledge rather differently: the one perhaps as a permanent black mark — the other as an open question about the future, not blind to risk but not fatalistic either.

More broadly, communication of risk concerns what people value and why; and especially how they regard the future. ‘The available research suggests that the response to uncertainty depends very much on the clinician's and patient's personal characteristics and values’ (Ahmed *et al.* 2012). This is always the case but especially perhaps in regard to matters of uncertainty — both about the way risk has been quantified (quality of underlying research) and as regards matters about which the risk is simply unknown. For example, religious belief in many people's lived experience shows up in what they value and how they regard the future, factors which in turn shape their interpretations of risk and their behaviour. How so? In the Psalm, self-knowledge is related to God's knowledge of the probability of any eventuality, and continuing fellowship amidst any eventuality: and therefore, perhaps, an assurance and perseverance in wise living amidst adversity.

Alternatively, some religious belief may give rise to cultural patterns that support fatalism, or even that genetic ill health is a kind of punishment from God. For example, some research around diabetes treatment in South Asian populations indicated that understanding religious beliefs about the origins of sickness may help in exploring resistance to screening and behaviour change (Choudhury *et al.* 2008; Watkins *et al.* 2013).

But religion is not all the same — there are differences *within*, for example, Islam, Christianity and Buddhism; and each involves quite different doctrines which shape self-knowledge: Jesus of Nazareth, for example, apparently rejected any association between wrongdoing by a person and conditions suffered by that person from the time of their conception and birth. Moreover, certain beliefs, benefitting from high-quality risk communication, may galvanise behaviour change. Alternatively, they may simply enable living *with* risk but *without* fear such as the belief that one's days are in God's loving hands as Psalm 139 suggests. Of course self-understanding is not simply religious. Engaging with how the *plurality* of belief and self-knowledge — religious or otherwise — actually shapes behaviour is the point being made. The influence of Jewish and Christian belief on self-knowledge makes that point particularly clearly but also signals the importance of conversation about the subtleties of belief in a plural society (Hordern 2016).

A challenge for personalised medicine may be that ‘knowing thyself cheaply’ fails to achieve the kind of cost reductions hoped for through prevention. One factor affecting this outcome is whether risk is related to people's values sufficiently to encourage preventative behaviour change. Any plausible effort to change people's behaviour must engage substantially and predominantly with people's cultures, values and beliefs and only about communication of genetic risk as a kind of catalyst for personal and social change (see also Horne, this volume).

## Self-knowledge and compassionate communion

The final observation is that self-knowledge is found in compassionate communion. Although culture, beliefs and values are the predominant modes of self-knowledge, stratification according to risk does offer an opportunity for a deepened solidarity amidst risky human life. This opportunity concerns *the ethos of stratification*: the psychological experience of being stratified with others according to a genetically defined understanding of a risk profile. Such an experience is an opportunity for self-knowledge of a particular kind: of knowing oneself as not being alone amidst the risks of life; of being joined together in therapeutic unity, activity and hope with other persons, whether complete strangers or family members.

Such unity in suffering or anticipated suffering can be profound if perhaps disturbing. It reflects, in Christian theological terms, the strange way in which God participates in human suffering through the death and resurrection of Christ, thereby creating communities of solidarity marked by compassion, joy and hope, what is called, in theological terms, communion. This is not a ‘holy’ compassion in the sense of the church's self-understanding which is unified in Christ but a tense communion in which many come together because a common concern but from different perspectives and with plural, pressing, urgent needs (Hordern 2014).

In such a tense communion of persons, even constrained by the risks genetically defined sub-groups face and even distinguished by the variety of personal narratives, needs and forms of self-knowledge, there can be freedom in fellowship amidst present and anticipated suffering. This sense of freedom is crucial to self-knowledge: that who I am and who we are is not determined by the risks we live with or the harms we may die from. Genetic solidarity groups formed in internet forums may or may not exhibit this kind of freedom, developing the ethos of stratification in concrete ways.

Such an ethos of stratification gives moral shape to efforts to tailor healthcare for specific sub-populations. It provokes further questions: how do people's plural values and beliefs shape participation in such a community? How does this affect adherence or concordance? Are there beneficial forms of psychological stratification on top of stratification according to genetic risk? Could these contribute towards motivating behaviour change, connecting risk perception with patient values? Further investigation of these questions will require collaboration across humanities, social science and medical science disciplines.

Of course, for some patients, behaviour change will have only a very small impact on their health; they need targeted therapies far more than a gym membership. The question is what positive moral interpretation could be made of the societal and clinical determination to focus on these citizens with such passion and excellence, when there are significant financial and opportunity costs to pursuing stratified medicine over against, say, public health initiatives focussed on behaviour change (Gaitskell, Gray et al., Sullivan and Gyawali, this volume).

Here is how the claim could run. *Stratified medicine offers a newly precise kind of humanising health care through deliberate, societal identification with the riskiest*.

To take a risk of theological interpretation, one might say that in terms of a parable Jesus is reported to have told, stratified medicine may reflect the work of the good Shepherd, who left the 99 behind, in order to locate and bring back the one sheep who was lost, who was least able to help themselves. The process of stratification not only leads to a connection between clinicians and those at most risk and most vulnerable but also, whether at the level of clinical trials or at the level of face-to-face/online support groups, between those patients themselves.

But lest this become too romanticised a view, one should observe that there are societal pitfalls here too. One German patient, for whom a specific targeted therapy was not likely to work, articulated her fear of becoming a ‘B patient’ (Wöhlke *et al.* 2015, p. 139), one of those who are molecularly unstratified and thus not ‘found’ in this sense. If such fears are reasonable, this may suggest that stratified medicine may accentuate the different paths of suffering and death along which people journey rather than drawing people together, thereby causing significant loss in terms of disappointment and unrealised hopes. This is a particular cause for concern requiring further research.

This patient's experience highlights the role of affections in risk communication: not only the role of affections in decision-making by cancer patients (Slovic *et al.* 2005) but also the significance of compassion more generally. Compassion understands the affections of another, seeking to participate and alleviate (Hordern forthcoming 2017). Compassionate clinicians need to be able to explore the right course of action in light of patient perceptions of risk *and* value, in terms of cultural, religious, philosophical and everyday beliefs; but also, more challengingly perhaps, to be able to communicate the futility of treatment in some circumstances, when the number of anyone's days comes into clearer focus.

Improving communication along these lines will require significant reserves of emotional depth on the part of clinicians and counsellors. Training and supporting staff in this way should therefore be a key dimension of the development of genomic medicine in the UK and elsewhere. This is the kind of compassionate communion we all need.

## Related video

A video linked to this paper can be found at: http://www.healthcarevalues.ox.ac.uk/joshua-hordern-personalised-medicine

## References

[CIT0001] Ahmed H., Naik G., Willoughby H. and Edwards A. 2012 Communicating risk. *British Medical Journal*, 344, e3996. doi: 10.1136/bmj.e3996 22709962

[CIT0002] Choudhury S., Brophy S., Fareedi M. A., Zaman B., Ahmed P. and Williams D. R. R. 2008 Intervention, recruitment and evaluation challenges in the Bangladeshi community: Experience from a peer lead educational course. *BMC Medical Research Methodology*, 8, 64. doi: 10.1186/1471-2288-8-64 18844992 PMC2571097

[CIT0003] Donnelly P. 2013 *The genetic revolution*. Available at: <http://www.well.ox.ac.uk/cpm/peter-donnelly-2> [Accessed 6 February 2017].

[CIT0004] Gigerenzer G. 2016 Full disclosure about cancer screening. *British Medical Journal*, 352, h6967. doi: 10.1136/bmj.h6967 26739799

[CIT0005] Heusser P. 2015 Towards integration of ‘personalised’ and ‘person-centred’ medicine: The concept of integrative and personalised health care. In Vollmann J., Sandow V., Wäscher S. and Schildmann J., eds. *The ethics of personalised medicine: Critical perspectives*. Farnham: Ashgate, pp. 77–83.

[CIT0006] Hollands G., French D., Griffin S., Prevost A. T., Sutton S., King S. and Marteau T. M. 2016 The impact of communicating genetic risks of disease on risk-reducing health behaviour: Systematic review with meta-analysis. *British Medical Journal*, 352, i1102. doi: 10.1136/bmj.i1102 26979548 PMC4793156

[CIT0007] Hordern J. 2014 Loyalty, conscience and tense communion: Jonathan Edwards meets Martha Nussbaum. *Studies in Christian Ethics*, 27(2), pp. 167–184. doi: 10.1177/0953946813514010

[CIT0008] Hordern J. 2016 Religion and culture. *Medicine*, 44(10), pp. 589–592. doi: 10.1016/j.mpmed.2016.07.011 PMC6320347 30636921

[CIT0009] Hordern J. forthcoming 2017 Compassion in primary and community healthcare. In Papanikitas A. and Spicer J., eds. *A handbook of primary care ethics*. Abingdon: Radcliffe/Taylor & Francis. 29300444

[CIT0010] Novas C. and Rose N. 2000 Genetic risk and the birth of the somatic individual. *Economy and Society*, 29(4), pp. 485–513. doi: 10.1080/03085140050174750

[CIT0011] Rehmann-Sutter C. 2010 Genes — cells — interpretations. In Pfleiderer G., Brahier G. and Lindpaintner K., eds. *GenEthics and**religion*. Basel: Karger, pp. 12–27.

[CIT0012] Slovic P., Peters E., Finucane M. L. and Macgregor D. G. 2005 Affect, risk, and decision making. *Health Psychology*, Jul:24(4 Suppl), pp. S35–S40. doi: 10.1037/0278-6133.24.4.S35 16045417

[CIT0013] UK Government Office for Science 2014 *Annual Report for the Government Chief Scientific Adviser. Innovation: Managing Risk, Not Avoiding It. Evidence and Case Studies*.

[CIT0014] Wäscher S., Schildmann J., Brall C. and Vollman J. 2015 ‘Personalised medicine’ in oncology: Physicians’ perspectives on contributions to and challenges for clinical practice. In Vollmann J., Sandow V., Wäscher S. and Schildmann J., eds. *The ethics of personalised medicine: Critical perspectives*. Farnham: Ashgate, pp. 149–159.

[CIT0015] Watkins Y., Quinn L. T., Ruggiero L., Quinn M. T. and Choi Y.-K. 2013 Spiritual and religious beliefs and practices, and social support's relationship to diabetes self-care activities in African Americans. *The Diabetes Educator*, 39(2), pp. 231–239. doi: 10.1177/0145721713475843 23411653 PMC3859187

[CIT0016] Wirth M. 2015 The authority of corporeality and emotions: The new phenomenology and its relevance to the German debate on personalised medicine. In Vollmann J., Sandow V., Wäscher S. and Schildmann J., eds. *The ethics of personalised medicine: Critical perspectives*. Farnham: Ashgate, pp. 65–75.

[CIT0017] Wöhlke S., Perry J. and Schicktanz S. 2015 Taking it personally: Patients’ perspectives on personalised medicine and its ethical relevance. In Vollmann J., Sandow V., Wäscher S. and Schildmann J., eds. *The ethics of personalised medicine: Critical perspectives*. Farnham: Ashgate, pp. 129–147.

